# Boiling histotripsy and in-situ CD40 stimulation improve the checkpoint blockade therapy of poorly immunogenic tumors

**DOI:** 10.7150/thno.49517

**Published:** 2021-01-01

**Authors:** Mohit Pratap Singh, Sri Nandhini Sethuraman, Craig Miller, Jerry Malayer, Ashish Ranjan

**Affiliations:** 1 Department of Physiological Sciences, College of Veterinary Medicine, Oklahoma State University, Stillwater, OK 74078.; 2 Department of Pathobiology, College of Veterinary Medicine, Oklahoma State University, Stillwater, OK 74078.

**Keywords:** Boiling histotripsy, αCD40, Checkpoint blockade, Cold tumors, Anti-tumor immunity

## Abstract

**Background:** Advanced stage cancers with a suppressive tumor microenvironment (TME) are often refractory to immune checkpoint inhibitor (ICI) therapy. Recent studies have shown that focused ultrasound (FUS) TME-modulation can synergize ICI therapy, but enhancing survival outcomes in poorly immunogenic tumors remains challenging. Here, we investigated the role of focused ultrasound based boiling histotripsy (HT) and in-situ anti-CD40 agonist antibody (αCD40) combinatorial therapy in enhancing therapeutic efficacy against ICI refractory murine melanoma.

**Methods:** Unilateral and bilateral large (~330-400 mm^3^) poorly immunogenic B16F10 melanoma tumors were established in the flank regions of mice. Tumors were exposed to single local HT followed by an in-situ administration of αCD40 (HT+ αCD40: HT40). Inflammatory signatures post treatment were assessed using pan-cancer immune profiling and flow cytometry. The ability of HT40 ± ICI to enhance local and systemic effects was determined by immunological characterization of the harvested tissues, and by tumor growth delay of local and distant untreated tumors 4-6 weeks post treatment.

**Results:** Immune profiling revealed that HT40 upregulated a variety of inflammatory markers in the tumors. Immunologically, HT40 treated tumors showed an increased population of granzyme B+ expressing functional CD8+ T cells (~4-fold) as well as an increased M1 to M2 macrophage ratio (~2-3-fold) and CD8+ T: regulatory T cell ratio (~5-fold) compared to the untreated control. Systemically, the proliferation rates of the melanoma-specific memory T cell population were significantly enhanced by HT40 treatment. Finally, the combination of HT40 and ICI therapy (anti-CTLA-4 and anti-PD-L1) caused superior inhibition of distant untreated tumors, and prolonged survival rates compared to the control.

**Conclusions:** Data suggest that HT40 reprograms immunologically cold tumors and sensitizes them to ICI therapy. This approach may be clinically useful for treating advanced stage melanoma cancers.

## Introduction

Immune checkpoint inhibitors (ICIs) targeting CTLA-4, PD-1, and PD-L1 proteins have revolutionized the treatment of melanoma and other tumor types in patients 1-4. Although promising, the immunosuppressive tumor microenvironment (TME) can influence ICI outcomes in a large proportion of treated patients 5-10. This occurs due to masking of tumor antigens and proliferation of suppressive immune cells (e.g., regulatory T cells and M2 macrophages), which directly influence the functions of cytotoxic T cells 11-15. Thus, there is a critical need to develop novel means for efficient activation of innate and adaptive immunity in the TME for superior ICI outcomes 16-20. Herein, we evaluated the role of anti-CD40 agonistic antibody (αCD40) combined with focused ultrasound (FUS)-induced boiling histotripsy (HT) in TME activation and ICI therapy of melanoma tumors.

Focused ultrasound (FUS) is a non-invasive treatment modality that utilizes sonic energy to treat at an unlimited depth from the body surface. We and others have shown that FUS thermal therapy has an immunomodulatory effect in melanoma tumors 21-23. However, tumor-heating approaches require accurate temperature feedback, and off-target effects can damage vital structures (e.g. nerves, blood vessels) adjacent to treated tissues. Recently, mechanical FUS was also shown to cause immune-modulations 24. In particular mechanical tissue homogenization using FUS based boiling histotripsy was found to be particularly efficient in enhancement of tumor inflammation 24-27, and anti-tumor immune effects 28, 29. HT can be attained by both cavitation or boiling means 30-33. However, boiling HT achieves instantaneous, predictable and controllable homogenization and heating of focal regions to 100 ºC by using millisecond-long pulses with lower pulse repetition frequency (PRF) 34. Boiling HT occurs for milliseconds, so it induces little or no damage to nearby structures due to minimal heat diffusion, while also achieving rapid accumulation of tumor-associated and neo-antigens in targeted tumor regions, enhancing immune cell infiltration by chemotaxis 35, 36. The activation of infiltrated antigen-presenting cells (APCs) and their subsequent migration to lymphoid tissues improve tumor antigen presentation to naïve T cells, thus causing antigen-specific tumor destruction 24, 37, 38.

Although the feasibility of HT in murine models has increasingly been reported 39, its ability to reprogram advanced stage poorly immunogenic tumors (e.g., B16F10) that lack major histocompatibility complex (MHC) and co-stimulatory molecules is not known. In general, “immunologically cold” tumors such as B16F10 exhibit minimal APC functions, failure to accumulate cytotoxic infiltrating lymphocytes, dominant expression of PDL1 on tumor cells, and poor response to ICIs in advanced stages, thereby evading antitumor immunity 40, 41. To overcome this barrier, recently we combined FUS-induced tumor heating (40-45 ºC) with in-situ αCD40 to achieve remissions of treated and untreated melanomas *in vivo* in a B16F10 mice melanoma model 21. We found that this combined treatment administered 3-4 times over 2 weeks increased the population and quality of T-cells (rich in Granzyme B and poor in PD-1 expression), and generated sufficient systemic antitumor immunity to significantly reduce growth of untreated contralateral tumors 21. αCD40 attaches to the CD40 receptor on APCs, enhancing CD40 signaling as well as expression of CD80, IL-12, and CCR7. These cause efficient APC activity and T cell-based cytotoxic responses 42-45. Since HT exerts its immunogenic actions by creating an *in-situ* depot of tumor antigens/debris and immunogenic cell death, with high spatial precision inside the tumor, and without significantly heating neighboring untreated tumor tissues, the objective of this study was to understand whether the local application of single HT and αCD40 (HT40) attains remission of advance stage melanoma tumors, addressing the current limitations of hyperthermia-based immunotherapy approaches. To investigate our hypothesis, we established late stage ICI refractory B16F10 melanoma and determine the mechanisms involved in APC infiltration and T cell homing with HT40 in treated and untreated remote tumors. Our data suggested that HT40 sensitized poorly immunogenic B16F10 melanoma to ICIs and improved the survival outcomes in melanoma bearing mice.

## Materials

B16F10 murine melanoma cells were provided by Dr. Mary Jo Turk at the Geisel School of Medicine at Dartmouth College (Hanover, NH, USA). They were cultured in DMEM supplemented with 10% fetal bovine serum and 1% streptomycin/penicillin. αCD40 (FGK45), anti-PDL-1 antibody (10F.9G2), and anti-CTLA-4 antibody (9H10) were purchased from BioXCell (West Lebanon, NH, USA). Fluorochrome-conjugated monoclonal antibodies (mAbs) purchased from BioLegend (San Diego, CA, USA) and BD Biosciences (San Jose, CA, USA) for flow cytometry were as follows: FITC, APC-Cy7 or PE-Cy7 anti-CD45.2 (104 and 30-F11), APC-Cy7 anti-CD11c (1A8), APC or BV786 anti-CD4 (GK1.5 and RM4-5), PE, PERCP, or BV510 anti-CD3 (145-2C11), BB515 anti-MHCII (2G9), PE anti-Granzyme B (QA16A02), APC anti-CD206 (C068C2), AF700 anti-IFN-γ (XMG1.2), BB700 anti-CD11b (M1/70), PE-Cy7 anti-IL-2 (JES6-5H4), APC anti-CD44 (IM7), AF488 anti-CD62L (MEL-14), BV711 anti-F4/80 (T45-2342), PE-Cy7 anti-CD8a (53-6.7), and Alexa Fluor 488 anti-Foxp3 (MF23). Quick-RNA Miniprep Kits were purchased from Zymo Research (Tustin, CA, USA). The nCounter PanCancer Immune Profiling Panel was purchased from NanoString Technologies, Inc. (Seattle, WA).

## Methods

### Mouse melanoma study design and ICI treatments

All the animal related procedures were approved by the Oklahoma State University Animal Care and Use Committee. For tumor inoculation, B16F10 cells at 80- 90% confluency were harvested, washed, and diluted with sterile cold PBS. Male C57/BL-6 mice (n=5/group, 6-8 weeks old), were subcutaneously implanted with 0.5 × 10^6^ cells (50 μL) in the right flank for flow cytometry and gene expression assessment. To measure the therapeutic effects (abscopal effect and survival rates), mice (n=5) were injected subcutaneously in the right flank on day 0 with 0.5 × 10^6^ cells and in the left flank on day 4 with 0.125 × 10^6^ cells 21, 46. Tumor volume of mice was measured every day using a serial caliper (General Tools Fraction™, New York, NY, USA); volumes were calculated using the formula (length × width^2^)/2, where length was the largest dimension and width was the smallest dimension perpendicular to the length. Treatments were initiated once the mice tumor volumes reached 330-400 mm^3^. For all studies, boiling HT treatment of tumors covered 40-50% of the tumor volume. αCD40 at a dose of 50 µg was injected by intratumoral injection within 2 h of HT. For pan-cancer immune profiling and flow cytometry, we compared the following groups: 1) Untreated Control, 2) HT, 3) αCD40, and 4) HT40. Mice tumors (n=3-5) and spleens (n=3-5) from surviving mice were harvested 1wk post treatment. For flow cytometry, harvested tissues were processed on the same day. For gene expression analysis, tumor tissues were snap-frozen in liquid nitrogen and stored at -80 °C until further use. For therapeutic assessments, we compared the following groups: 1) Untreated Control, 2) HT, 3) αCD40, and 4) HT40, each with and without the combination of anti-CTLA-4 and anti-PDL-1. Anti-CTLA-4 (100 µg/dose) and anti-PD-1(200 µg/dose) were injected intraperitoneally following HT, αCD40, or HT40 treatment, and two subsequent ICI dose were given every third day. Mice were sacrificed for survival studies when the tumors reached ~2 cm in any dimension.

### Boiling HT set-up and tumor exposures

We utilized the Alpinion FUS transducer with a 1.5 MHz central frequency, 45 mm radius, and 64 mm aperture diameter with a central opening of 40 mm in diameter for HT exposures. For ultrasound exposure, the tumor was aligned at a fixed focal depth to cover voxel size of 1 x 1 x 10 mm. VIFU-2000 software was used to define the target boundary and slice distance in x, y, and z directions for automatic rastering of the transducer during treatment. The focal points were rastered to cover 40-50% of the tumor. HT was performed by using boiling histotripsy regimen (1 Hz PRF, 1% duty cycle, 450 W acoustic power) and were adapted from prior publications that used a similar device 39, 47. Each focal spot was treated for 10 s. Mice were administered buprenorphine (0.05 mg/kg; SC) daily for 3 days post HT treatment.

### Histopathological analysis of treated and untreated tumors

HT was confirmed by histopathology. HT exposed tumor tissues (n=3) were fixed in 10% neutral buffered formalin, processed, and embedded in paraffin 48. Histopathological examinations of 4 μm sections stained with hematoxylin and eosin (H&E) were performed by a veterinary pathologist. To examine the tumor immune environments, the tumor sections from mice bearing bilateral tumors were stained for CD3 antibody for immunohistochemical analysis. Briefly, 10% neutral buffered formalin fixed treated and untreated (abscopal) tumors were embedded in paraffin and cut into 4 μm sections. Antigen retrieval was performed in a decloaking chamber. Normal goat serum was used for blocking. Incubation with the anti-rat CD3 primary antibody (Abcam, Cambridge, MA) was performed at 4°C overnight. Biotinylated goat anti-rat (Vector Laboratories, Burlingame, CA) was used as the secondary antibody and detected with ImmPACT DAB HRP Substrate (Vector Laboratories, Burlingame, CA).

### Pan-cancer immune profiling of tumors

Total RNA extracted from snap-frozen tumors (n = 3/group) using the Quick-RNA Miniprep Kit (Zymo Research) was profiled using the nCounter® PanCancer Immune Profiling Panel (NanoString Technologies, Inc., Seattle, WA, USA). This panel contains 770 genes involved in the cancer immune response. Gene expression profiling was performed using the following steps: (i) Hybridization: 25 ng of total RNA were hybridized with the mouse PanCancer immune profiling code set having 770 unique pairs of 35-50 base pair biotin-labeled capture probes and reporter probes with internal reference controls. Hybridization was performed overnight at 65 °C. (ii) Washing: Excess probes were removed with magnetic bead purification on the nCounter® Prep Station (software v4.0.11.2). Unbound probes were washed away, the tripartite structure was bound to the streptavidin-coated cartridge by the biotin capture probe, aligned by an electric current (negative to positive), and immobilized. Degradation of fluorophore and photobleaching were prevented by adding SlowFade. Read counts from the raw data output were assessed for differential gene expression and cell type scoring after normalization using NanoString nSolver (version 3.0) 49. Briefly, Log_2_ counts were represented as z-scores in heat map to indicate alterations in gene expression and immune cell profile for each sample. Additionally, the relative differences in gene signatures between treated and control tumors were represented as volcano plots (log_2_ fold change vs log_10_ P-value).

### Immune profiling of melanoma tumors by flow cytometry

Tumors were mechanically disrupted and digested with 200 U/mL collagenase IV (Life Technologies, NY, USA) followed by filtration through a 70 μm cell strainer (Corning Inc., Corning, NY, USA) to obtain a single cell suspension. Fixable Viability Stain 575V (BD Biosciences) was used to stain cell suspensions to exclude dead cells from analysis as per the manufacturer's instructions. To block FcγIII/II receptor-mediated unspecific binding, anti-CD16/CD32 antibody was used. Cells were stained with indicated anti-mouse fluorochrome-conjugated antibody combinations for 30 min on ice in the dark using the following panel: CD45+ (tumor infiltrating leukocytes; TILs), CD11b+, F4/80+ (macrophages), CD11b+, F4/80+, MHCIIhi (M1 macrophages), CD11b+, F4/80+ MHCII lo/neg, CD206+ (M2 macrophages), CD11b+ CD11c+, F4/80-, MHCII+ (dendritic cells), CD3+, CD4+ (CD4+ T or helper Th cells), CD3+, CD4+, CD44hi CD62lo (CD4+ T effector/memory cells), and CD3+, CD8+ (CD8+ T cells). To detect IFNγ, IL-2, Granzyme-B, and Foxp3 positive T cells, cells (without stimulation) were washed after surface marker staining, fixed and permeabilized with a transcription factor buffer set (BD Biosciences), and incubated with Pe-Cy7 anti-IL-2, BV650 or APC-Cy7 anti- IFNγ, PE anti-Granzyme-B, or Alexa Fluor 488 anti-Foxp3 antibody for 30 min in the dark on ice 23, 46, 50. Stained cells were run in an LSRII flow cytometer (BD Biosciences) within 24 h. Compensations were performed with single-stained UltraComp eBeads or cells. FlowJo software v.10.2 (Treestar Inc., Ashland, OR, USA) was used for data analysis. For all channels, positive and negative cells were gated based on a fluorescence minus one control.

### Evaluation of the melanoma-specific systemic T cell response

Single cell suspension of splenocytes were stimulated ex-vivo with the melanoma-specific differentiation antigen tyrosinase-related protein 2 (TRP-2) peptide for 8 h to determine generation of TRP-2 melanoma antigen specific immunity in mice 51, 52. Briefly, 1-2 x 10^6^ splenocytes were incubated at 37 °C and 5% CO_2_ with 2.5 µg of TRP-2 peptide for 8 h in the presence of Brefeldin A (eBioscience, San Diego, CA; 1000X solution). Treated cells were washed with PBS and stained with CD45, CD3, CD4, CD8, IFNγ and IL-2 antibodies for flow cytometry analysis. The number of T effector cells responding to TRP-2 stimulation was calculated as CD45+ CD3+ CD4+ or CD8+ T cells that were positive for IFNγ or IL-2, and results were expressed as percentage of total splenocytes.

### Tumor regression and survival rate evaluations in murine melanoma

Tumor regression in the treated and untreated sites were determined by computing the difference in the tumor volumes for the various groups relative to untreated control. For survival studies, tumor bearing mice were followed for 40 days post inoculation, and the median survival for each treatment group was assessed by the Kaplan-Meier survival curve.

### Statistical analyses

Statistical analyses were performed using GraphPad Prism 8.4.2 software (GraphPad Software Inc, La Jolla, CA, USA). The differences between the treatments compared to the untreated control were analyzed by multiple t-tests without multiple comparisons correction. The nanostring data were represented as mean of log_2_ fold change relative to control. All other data were presented as mean ± standard error of the mean (SEM) unless otherwise indicated. For analysis of three or more groups, one-way analysis of variance was performed followed by Tukey's multiple comparison tests. The overall P value for Kaplan-Meier analysis was calculated using the log-rank test. Analysis of differences between two normally distributed test groups was performed using an unpaired t-test assuming unequal variance and multiple t-tests. P < 0.05 was considered to be statistically significant.

## Results

### Local HT achieved precise homogenizations of the treated regions

H&E showed that HT created a core of homogenized tumor tissue covering 40-50% of the total volume and this was surrounded by intact tumor tissue (Figure 2A). There was a clear transition zone between the HT-treated and non-treated tumor regions such that viable tumor tissue was negligible in the area treated with HT. These were also verified by real-time US imaging during HT treatment in those regions, whereby hyperechoic regions during each pulse at the focal point followed by hypoechoic contrast at the end of the pulse was noted (Figure 2B-D).

### HT40 induced inflammation and checkpoint expression in established melanoma

**Figure 1:**
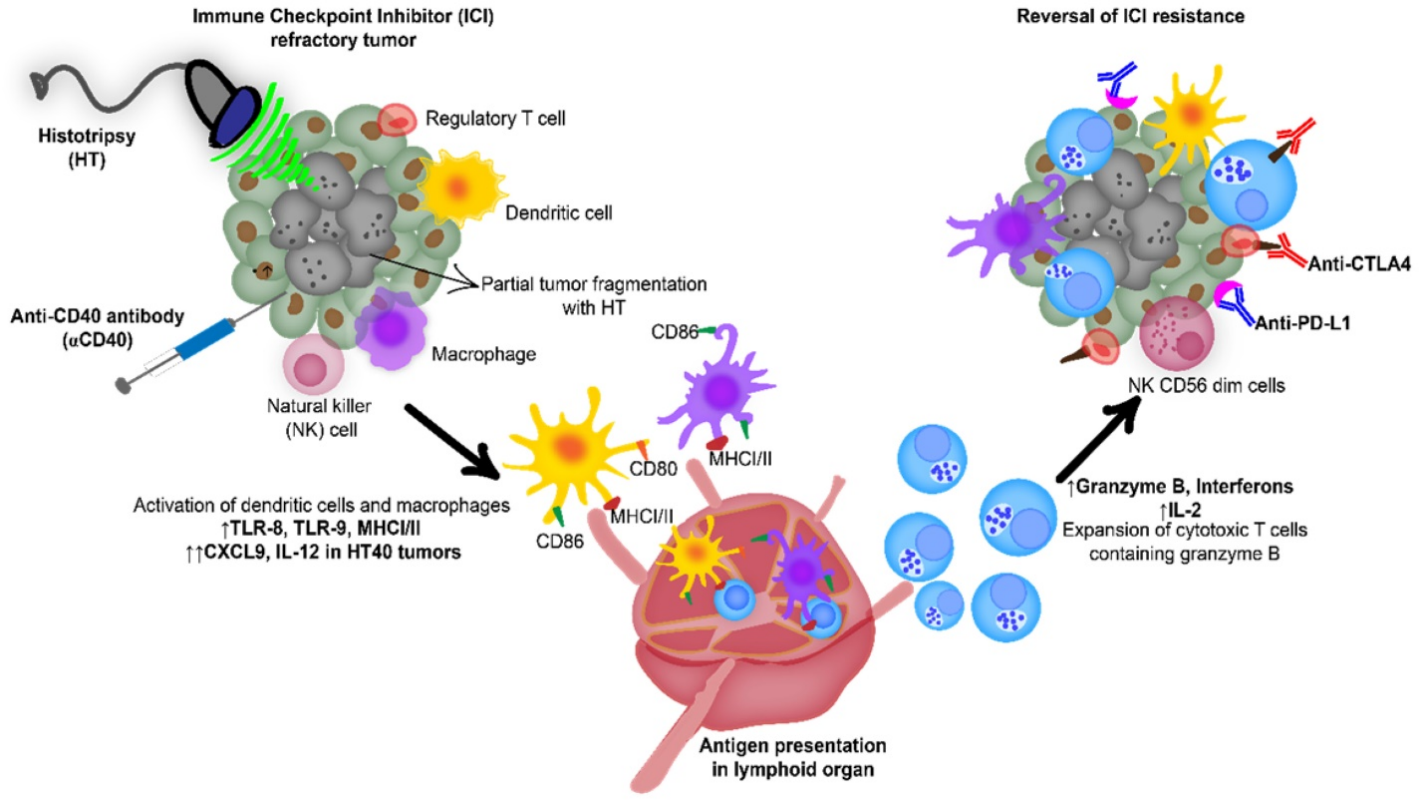
Combination of anti-CD40 agonistic antibody (αCD40) with focused ultrasound (FUS) induced boiling histotripsy (HT) activates the tumor microenvironment to enhance the efficacy of immune checkpoint inhibitors in poorly immunogenic tumors.

HT40 was performed in B16F10 melanoma tumors established unilaterally (Figure 3A). Screening of immune related genes (n=3/treatment group) in the tumor microenvironment using nanostring technique suggested an increased expression of inflammatory genes associated with phagocytosis, cell adhesion, cytokine, and antigen processing and presentation for HT, αCD40 and HT40 compared to the control, but this profile was most significant and dominant in HT40-treated tumors (Figure 1 and S1). HT alone increased immune infiltration markers (1.26 log_2_ fold for ICAM-2 and 0.71 log_2_ fold for VCAM-1), and APC chemo attractants (CCL8: ~2.6- and CSF1R: ~1.78-log_2_ fold) compared to control (Figure 3B; also see Figure S2 volcano plots for quantitative changes in gene expression). αCD40 and HT40 upregulated the expressions of the genes associated with CD45, T cells, and NK cell activations (Figure 3C). Also, HT40 tumors had enhanced dendritic, Th1, CD8+ T, cytotoxic, and NK CD56^ dim^ cell markers. HT40 increased the expression of CXCL9 (~4.23 log_2_ fold), TLR-8, TLR-9 (~2 log_2_ fold), IL12-α and STAT1 (~1 log_2_ fold) genes (Figure 3B). Further, it upregulated the T cell activation genes (IFNβ1, IFNL2, granzyme α, granzyme β, IL1b, IL2, ICOSL, ICOS, TBET, CD69, CD44, CD160, and 4-1BB) and downregulated TGFβ2 (Figure 4A). Consistent with T cell activation, the checkpoint marker genes (CTLA4, PDL1, PD1, TIM3, and LAG3) were enhanced with αCD40 and HT40 treatment (Figure 4B). In particular, immune activation markers such as TIGIT, IDO1, STAT1, and EOMES were significantly expressed in HT40-treated tumors relative to controls (Figure 4B). Finally, to test, whether the gene expression results correlated with flow cytometry findings, we isolated the CD45+ and CD45- cells harvested from the tumors and assessed the PDL1 expressions. A 1.3-1.5-fold enhanced expression of PDL1 in TILs for αCD40 and HT40 treated tumors were noted, demonstrating strong associations between assays (Figure 4C).

### Local treatments suppressed tumor progression by enhancing melanoma immunogenicity

Mice with unilateral B16F10 tumors in the flank regions were established. HT treatment alone slightly inhibited the tumor growth rate 1-week post treatment, but its combination with anti-CD40 antibody reduced tumor growth by > 70% compared to the control. This reduction was 30-50% greater than that of respective monotherapies (Figure 5A). The reduction in tumor volumes accompanied a significant reduction in tumor weights for the HT40 cohort compared to the other groups (Figure 5B). Local and systemic evaluation of the immune responses of harvested tumors from the surviving mice revealed an increase (~1.2-2-fold) in the populations of CD45+ TILs and CD3+ T cells in the HT-treated group compared to the untreated control. The TIL increase was not accompanied by a significant increase in CD8+ subtypes in HT-treated tumors. In contrast, HT40 enhanced the CD3+ CD8+ T cell population by 2-3-fold relative to HT post treatment (Figure 5C-E). The populations of effector CD8+ T cells exhibited an increased level of IFNγ and granzyme B expression, suggesting an activated cytotoxic phenotype (Figure 6A-B). We also found that the T cell activation was not accompanied by a concurrent increase in the Foxp3+ CD4+ Tregs. Overall, a 2.5 to 5-fold increase in the granzyme B+ CD8+ T cell to Treg ratio in αCD40 and HT40-treated tumors compared to the untreated control was noted, which reflects enhanced mobilization of cytotoxic cells in the treated tumor (Figure 6C).

### HT40 promoted melanoma specific immunological memory

A significant increase in CD44+ CD62lo CD4+ T cells, which represent the CD4+ effector-memory T cell population, was observed for the HT- and HT40-treated tumors (1.5-2-fold). Additionally, an increased population of M1 macrophages along with a concurrent decrease of M2 macrophages was noted for HT40-treated tumors. αCD40 alone did not increase CD4+ effector cells, but it did enhance the populations of M1 macrophages, which suggested APC activation (Figure 7A-C). HT, αCD40, and HT40 also increased M1 macrophages and reduced the M2 phenotype in the spleen tissues, with the HT40 having the greatest effect (Figure 7D-E). To assess antigen specificity, splenocytes stimulated ex vivo with TRP-2 were assessed for IL2 production. A significant (1.3-1.7-fold) increase in TRP-2 specific IL2+ CD4+ T cells in the spleen of HT40-treated mice compared to the control was noted, and this number was relatively higher compared to that of the other therapies (Figure 7F). Thus, we posited that the HT40 induced a potent melanoma memory response.

### HT40 therapy sensitized mice bearing bilateral melanoma tumors to Immune checkpoint inhibitors

B16F10 melanoma are known to demonstrate a modest response to ICIs 53, 54. Also, ICIs are most effective when administered immediately post tumor inoculation 55. Since the goal of this study was to assess the feasibility of our combinatorial approach in immune-resistant tumors, we performed treatments in B16F10 tumors when they reached a volume of 330-400 mm^3^. For assessing ICI effect in a bilateral melanoma model, unilateral HT40 treatment of the right flank tumor was followed by intraperitoneal injection of ICIs (n=5 per group, Figure 8A). ICI by themselves were ineffective in inducing tumor growth suppression and survival rates compared to the control, suggesting that the B16F10 melanoma was refractory to the checkpoint blockade therapy at the time point tested (Figure 8B and Figure 9). Also, HT or αCD40 alone moderately enhanced ICI efficacy and survival compared to ICI and untreated controls. In contrast, HT40 significantly improved outcomes *vs*. HT or αCD40 alone. Also, when primed with HT40, ICI therapy was most effective in delaying tumor growth rates, and in enhancing survival responses *vs*. all other treatments. In general, untreated control mice that were bilaterally inoculated didn't survive beyond day 23 post inoculation, suggesting an absence of abscopal effect. For other treatments (especially for the HT40), the presence of abscopal effect induced a prophylactic effect, enhancing the survival rates presumably by decreasing the overall tumor burden in the treated mice (Figure 9). We found that 40% (2 of 5) of HT40+ICI-treated mice showed abscopal tumor suppression for the entire treatment period (40 days; not shown). In contrast, other treatments were relatively less effective, and mice reached euthanasia endpoints before the end of study. To understand the mechanisms, and examine the change in tumor microenvironments in treated and untreated tumors, we processed both treated and abscopal tumors for CD3 immunohistochemical analysis. The treated and untreated tumors from HT and αCD40 alone showed mild T cell infiltration. In contrast, the treated and untreated HT40 tumors showed a prominent increase in the density of CD3+ T cells within the tumor compared to monotherapies (Figure 10), correlating with the improved therapeutic outcomes in HT40 cohorts.

## Discussion

The objective of this study was to understand the ability of HT40 to reprogram the immunologically cold melanoma tumor such that it becomes more receptive to ICI therapy. HT has been utilized to debulk tumor tissue, release damage associated molecular patterns (DAMPs), and improve immune sensitization in various tumor models 24, 27, 36, 56. We and others have also shown that local αCD40 therapy activates APCs and improves the functional status of TILs in melanoma 21, 57, 58. This is likely via enhanced antigen presentation by APCs through improved CD40L binding with CD40 receptor on APCs, and by the upregulation of costimulatory molecules such as MHC class II, CD80, CD86, and CD58 on the cell membrane 59. Particularly, we have found that that αCD40 efficacy is enhanced with local FUS-heating of B16F10 melanoma in mice 21. Tumor heating is highly challenging to perform in highly perfused organs, and can cause collateral damage to nearby healthy tissues 60. To overcome this barrier, in this study we combined boiling HT and αCD40 for anti-tumor immunity induction in immunologically cold melanoma tumors. Boiling HT is a non-invasive mechanical homogenization technology that rapidly generates tumor antigen depots with sharp boundaries in solid cancers. Since HT achieves heating to 100 ºC by using millisecond-long pulses with lower pulse repetition frequency (PRF) 34, it avoids denaturation of tumor antigens in the focal regions 36. Thus, its combination with αCD40 can hypothetically improve tumor immune environment, and immunotherapeutic response.

**Figure 2:**
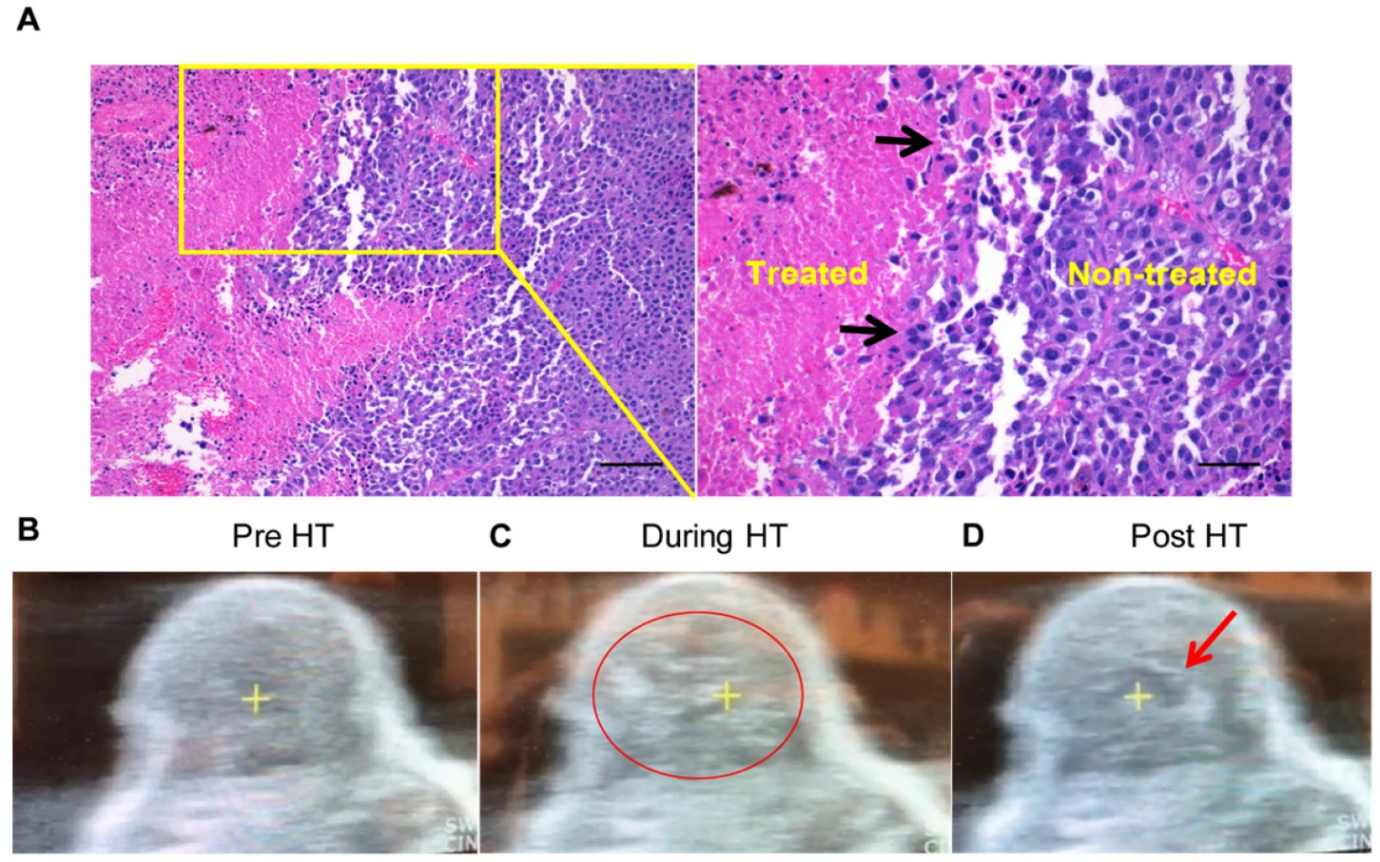
** Local HT achieved precise melanoma homogenizations.** (A) The H&E stained tumor sections showing sharp transition zone (black arrows) between histotripsy treated and untreated tumor region (n=3). Scale bar: 200μm (left image) and 100μm (right image). (B-D) HT therapy induced homogenizations of treated tumor regions (B) Pre-treatment image of a mouse tumor. (C) HT treatment produced hyperechoic regions during each pulse (indicated by the red circle). (D) Hypoechoic contrast at the end of the pulse was visible adjacent to the focal point (indicated by red arrow).

**Figure 3:**
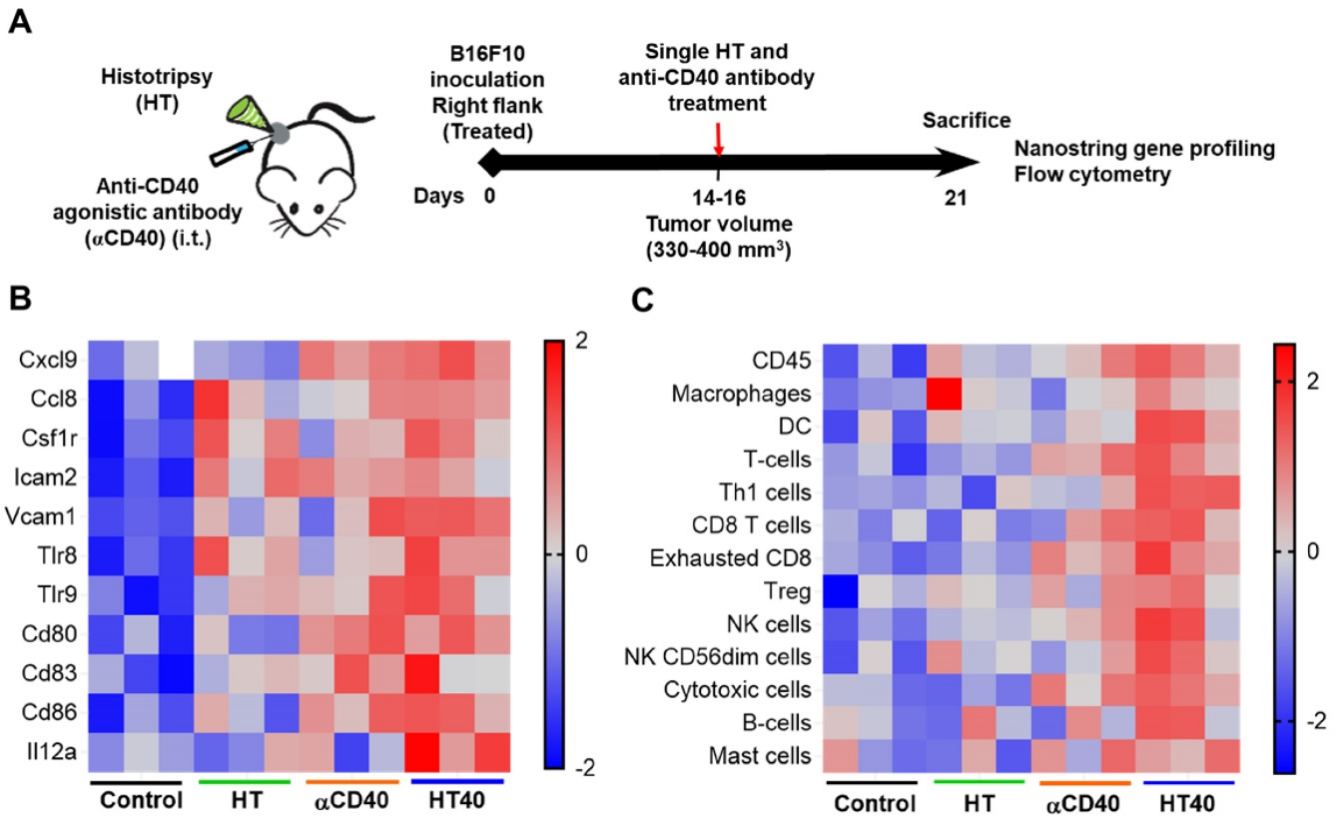
** HT40 therapy increased pro-inflammatory immune markers in tumors.** (A) C57BL/6J mice were implanted subcutaneously in the right flank with B16F10 cells unilaterally and single treatments of HT, αCD40 or HT40 were administered (n=5 per group). Tumors were harvested 7 days post treatments. Total RNA (n = 3 samples per treatment group) was isolated, and immune profiling was performed using the NanoString PanCancer Immune panel. (B) Heat maps showing gene markers of cell adhesion molecules, chemokines, innate sensors, and activation status of APCs was higher for HT40 tumors relative to the corresponding controls. (C) Total tumor infiltrating leucocytes, dendritic cells, Th1 cells, cytotoxic cells and activated NK cell expression markers were significantly higher with HT40 therapy compared to the controls. Please see supplemental figure S2 for mean log2 fold changes for individual gens compared to the control. Statistical analysis was performed using multiple t-tests without correction for multiple comparisons. p < 0.05 is considered significant.

**Figure 4:**
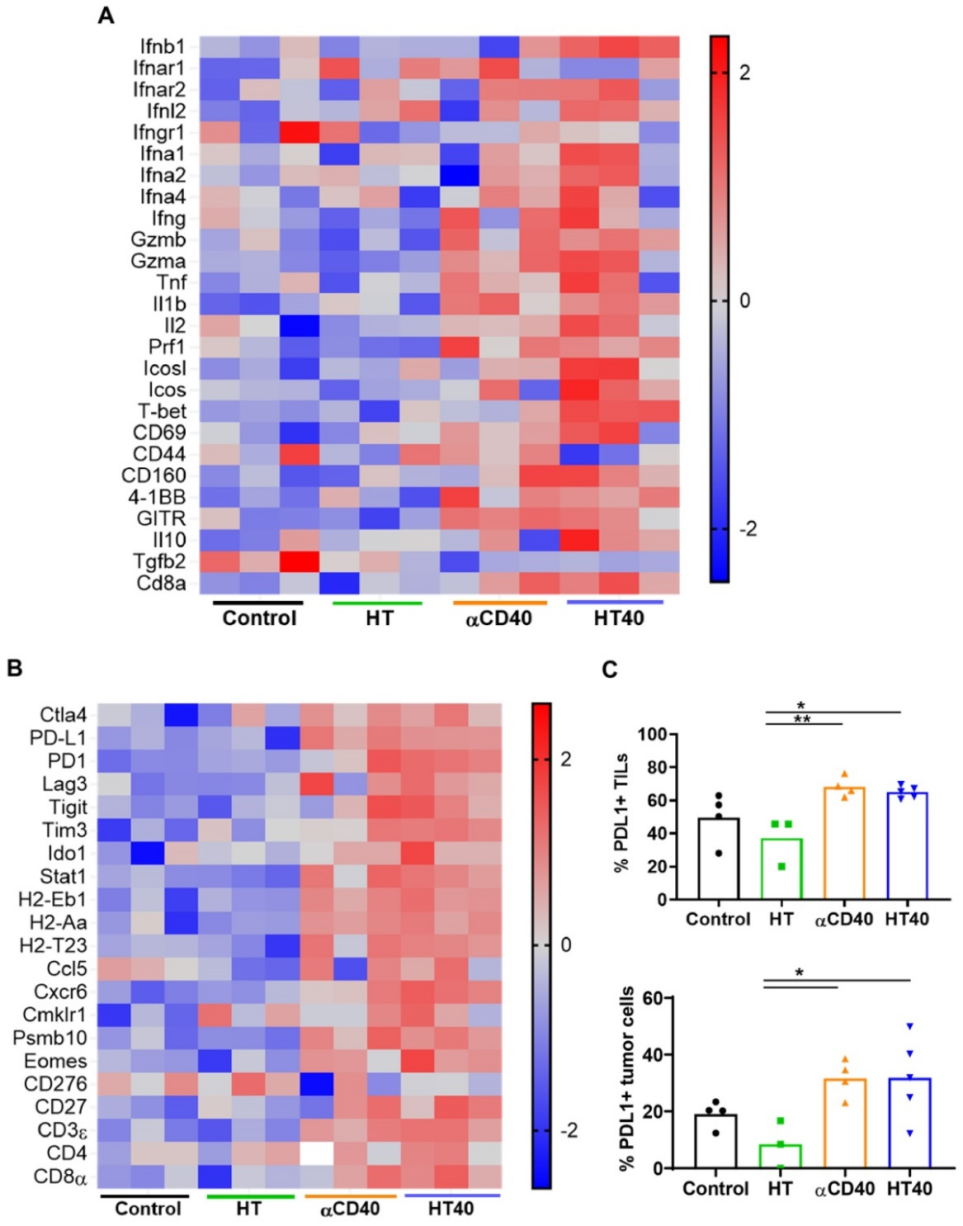
** HT40 and αCD40 therapy enhanced T-cell activation and checkpoint expressions in the melanoma tumors.** (A) Heat maps showed an enhanced expression of T-cell activation genes in the treated tumors compared to the control. (B) The checkpoint marker genes (e.g. CTLA4, PDL1, PD1, TIM3, and LAG3) were enhanced with CD40 and HT40 treatment. (C) PD-L1+ CD45+ (tumor infiltrating leukocytes; TILs) and PD-L1+ CD45- (tumor cells) cells assessed using flow cytometry (n=3-5 per group). Gene expression statistical analysis was performed using multiple t-tests without correction for multiple comparisons (n=3 per group). p < 0.05 is considered significant. For flow cytometry (C), data were presented as mean ± SEM and the statistical differences between groups were measured by ANOVA followed by Tukey's multiple comparisons. * p < 0.05, ** p < 0.01.

To investigate the potential of the HT and αCD40 combination, we utilized an ICI refractory and poorly immunogenic B16F10 model. B16F10 tumors exhibit reduced expression of the MHC class I and co-stimulatory molecules (e.g. CD80, CD86 etc.) 40. Its self-antigen (TRP-2) also shows poor affinity to T cell receptors, thereby making it an excellent poorly immunogenic model for immunotherapy studies 61, 62. High intensity, low duty cycle, and short ultrasound HT pulses were used to fractionate ~40-50% of the tumor mass (Figure 2A-B). Pan-cancer immune profiling suggested that the selected HT parameters elevated the expression of chemo-attractants (CCL8 and CSF1R) and cell adhesion molecules (ICAM and VCAM). These markers are essential for cell-cell interaction and leukocyte migration into tumors (Figure 3) 63-65.

**Figure 5:**
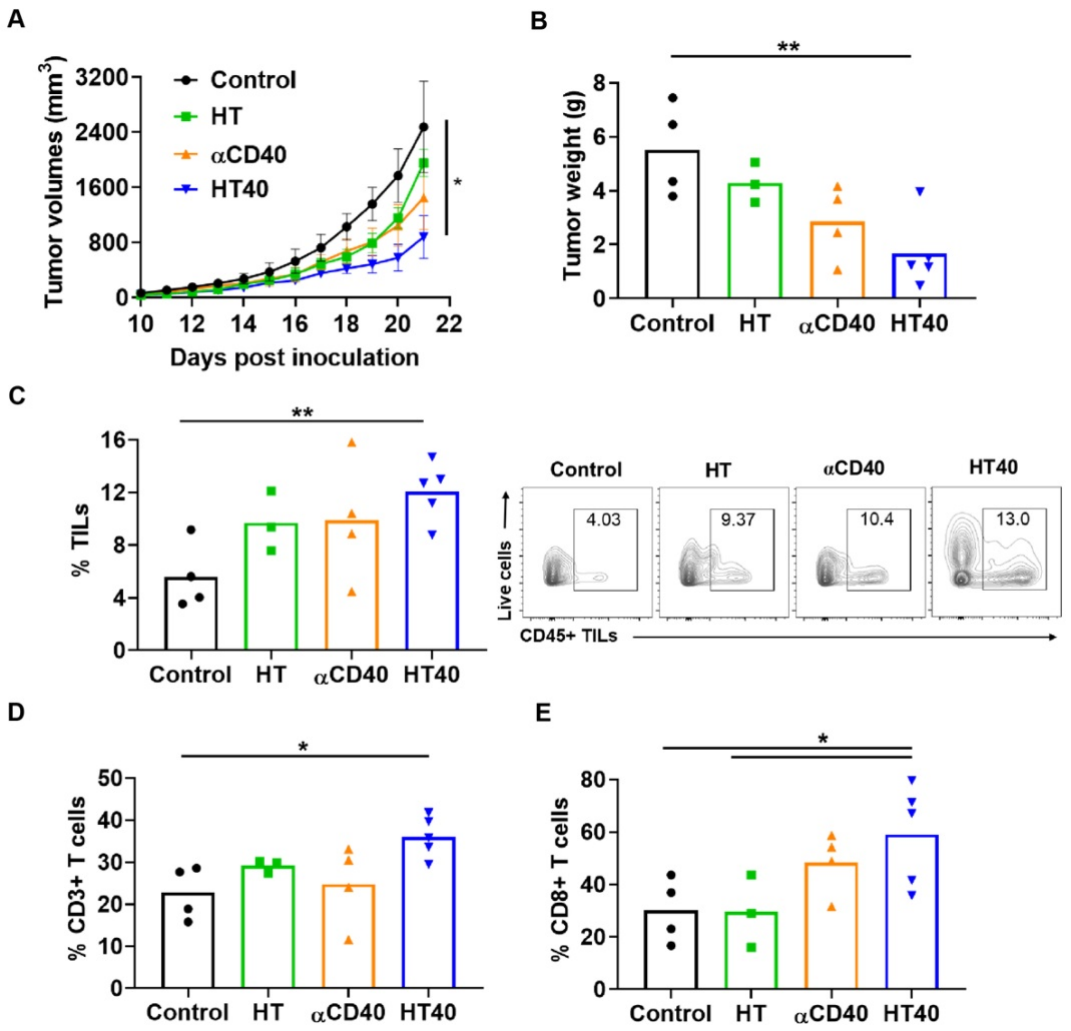
** Local HT40 suppressed tumor progression and improved the infiltration of T lymphocytes.** (A) Time course of treatment effects on tumor volumes through 21 days post-inoculation and (B) Tumor weights at time of harvest in mice unilaterally inoculated with B16F10 cells in the right flank regions. HT40 inhibited tumor growth significantly *vs*. that of the respective controls (C) HT, αCD40, and HT40 enhanced the populations of tumor infiltrating leucocytes (TILs) compared to control in the harvested tumors of surviving mice. Overall, HT40 demonstrated the highest infiltration rates compared to the other groups. (D) HT40 induced a higher percentage of CD3+ T cell population than the control. (E) Frequency of CD8+ T cells in HT40 group was 2-folds higher compared to the HT and control group. Results are shown as mean ± SEM, n=3-5 per group. One-way ANOVA followed by Tukey's multiple comparison was used for data analysis. * p < 0.05, ** p<0.01.

**Figure 6:**
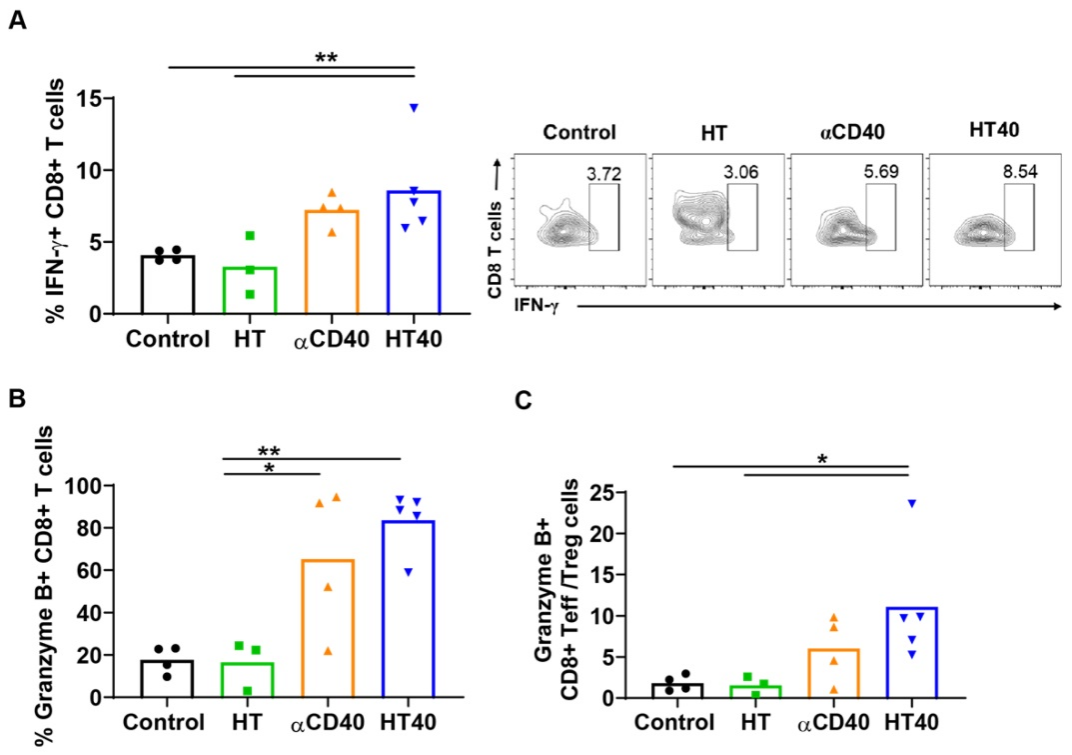
**HT40 augmented the T cell functions in treated tumors.** (A and B) HT40 promoted IFNγ (~2-fold) and Granzyme B (~4-fold) secretion from CD8+ T cells in the treated tumors. (C) Ratio of cytotoxic CD8+ T cells and immunosuppressive regulatory T (Treg) cells in tumors increased by 2.5 and 5-fold with αCD40 and HT40 compared to the untreated controls, respectively. Data are shown as mean ± SEM, n=3-5 per treatment group, * p < 0.05, ** p < 0.01. Data were analyzed by One-way ANOVA followed by Tukey's multiple comparisons; changes between control and treatments in Figure 6C were analyzed using an unpaired t test assuming unequal variance.

**Figure 7:**
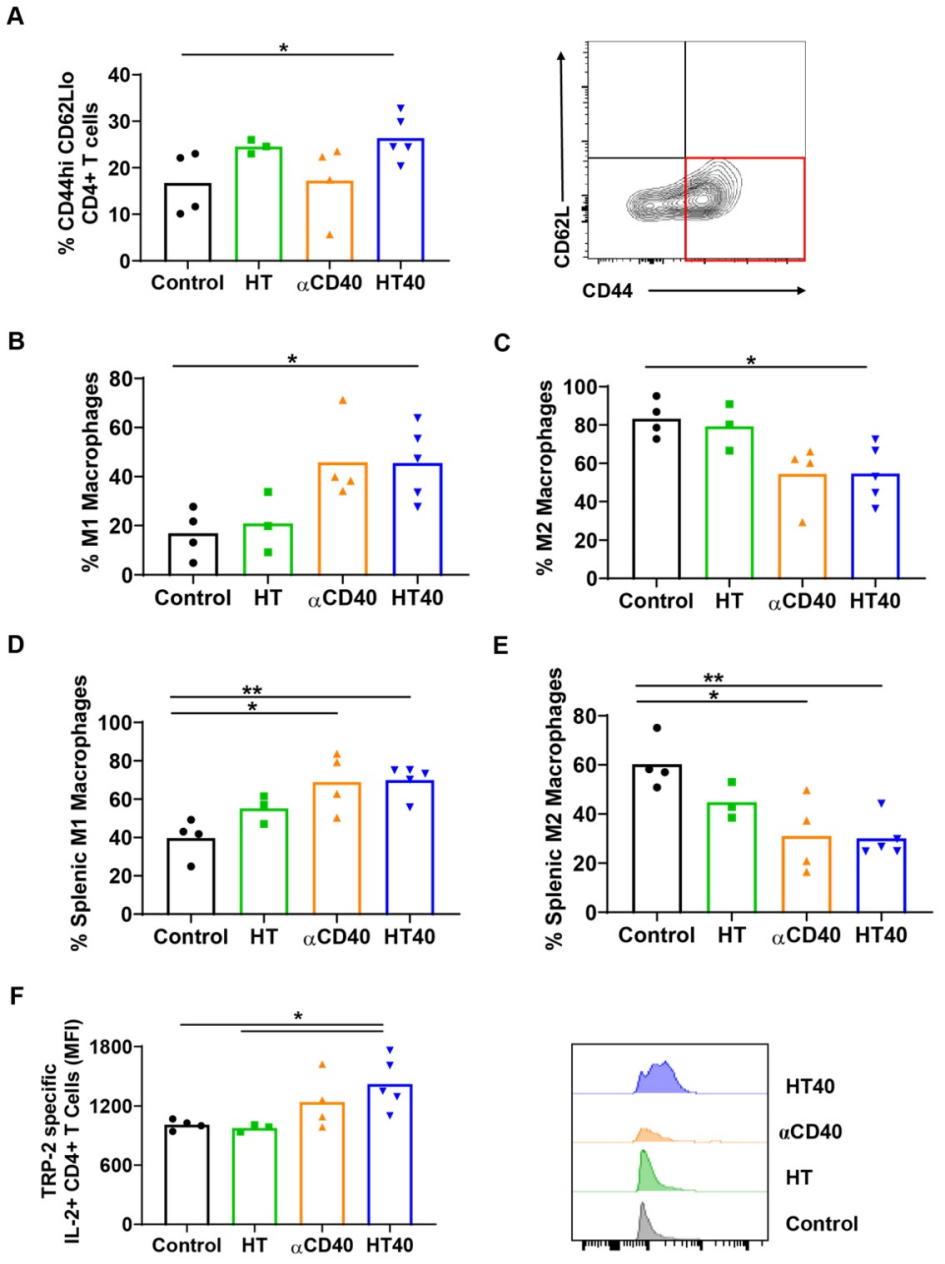
** HT40 increased melanoma specific antitumor immunity.** (A) A significant increase in CD44hi CD62lo CD4+ effector T memory cells (percentage out of total leukocytes) in HT and HT40 treated tumors was noted. (B and C) αCD40 and HT40 enhanced the percent of M1 macrophages by 2-fold and decreased M2 macrophages by 1.5-fold compared to controls. (D and E) HT, αCD40 and HT40 increased M1 macrophages (~1.3-1.7-fold) and decreased M2 macrophages (~1.5-2-fold) in splenic tissues compared to the control. (F) IL-2 production from CD4+ T cells was significantly improved by αCD40 and HT40 treatments compared to untreated controls. Amongst all the treatments, HT40 showed the most dominant effect upon TRP-2 stimulation *ex-vivo*. Data are shown as mean ± SEM, n=3-5 per group. Data were analyzed by ANOVA followed by Tukey's multiple comparisons. * p < 0.05, ** p < 0.01.

**Figure 8:**
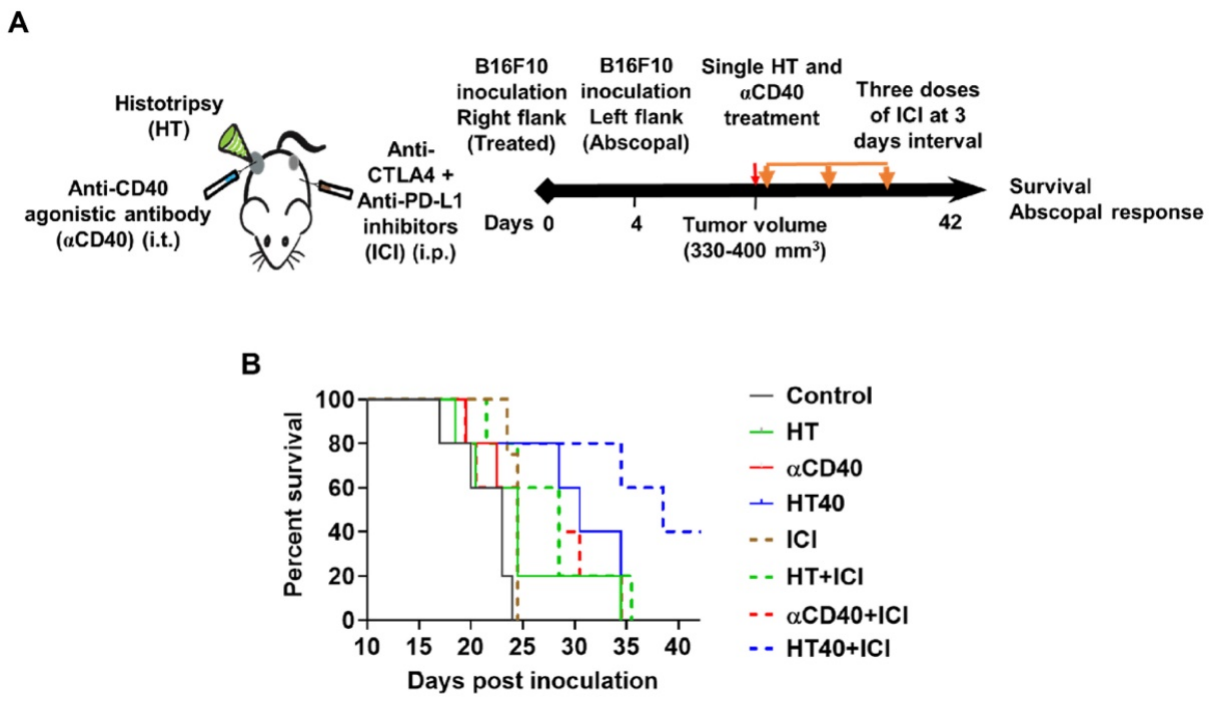
**HT40 priming enhanced the therapeutic effects in ICI refractory melanoma.** (A) Mice bearing B16F10 melanoma in the left and right flank regions were treated unilaterally followed by ICI therapy. (B) ICI combinations alone were ineffective in enhancing survival rates *vs*. those of controls, suggesting that at the time-point tested, the B16F10 melanoma was refractory to ICIs. HT40 improved ICI survival outcome compared to other groups by reversing resistance to ICIs. Differences in median survival (n=5) were determined by the Kaplan-Meier method and the log-rank test used to determine P value. Differences in the median survival (n=5 per group) were determined by the Kaplan-Meier method and the log-rank test was used to determine P value. p < 0.05: HT40+ICI vs αCD40+ICI; p < 0.1: HT40+ICI vs HT+ICI, HT40.

**Figure 9:**
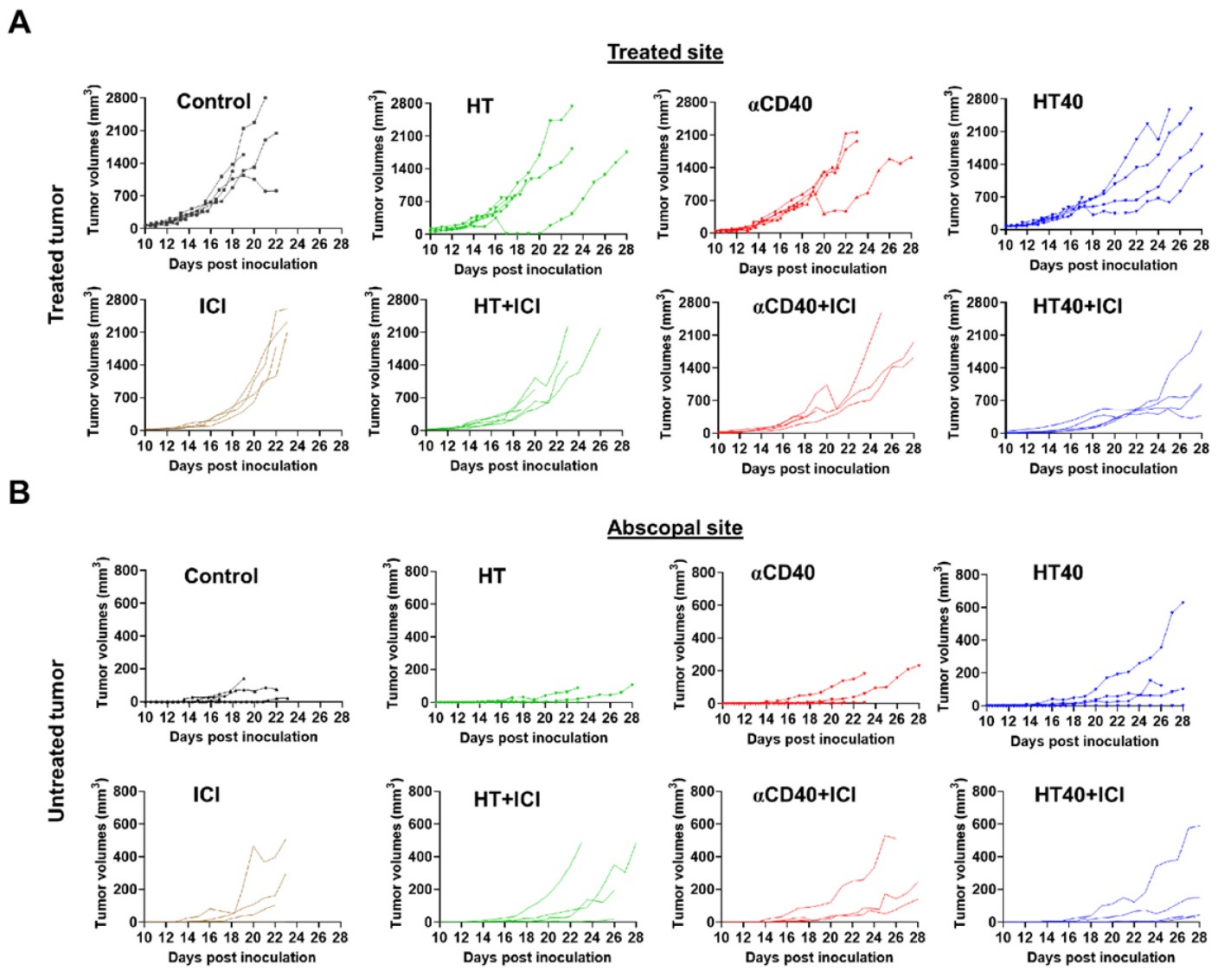
** Tumor growth rates in mice bearing melanoma in left and right flank regions shown till day 28 post inoculation.** (A) HT40 and HT40+ICI delayed growth of treated tumors compared to HT and αCD40 alone. (B) Tumor growth rates at distant untreated sites were relatively slower with HT40+ICI and HT40 compared to other treatments. Untreated and HT treated mice succumbed to the disease between day 22-26, preventing the estimation of tumor growth rates at the untreated/abscopal sites.

**Figure 10:**
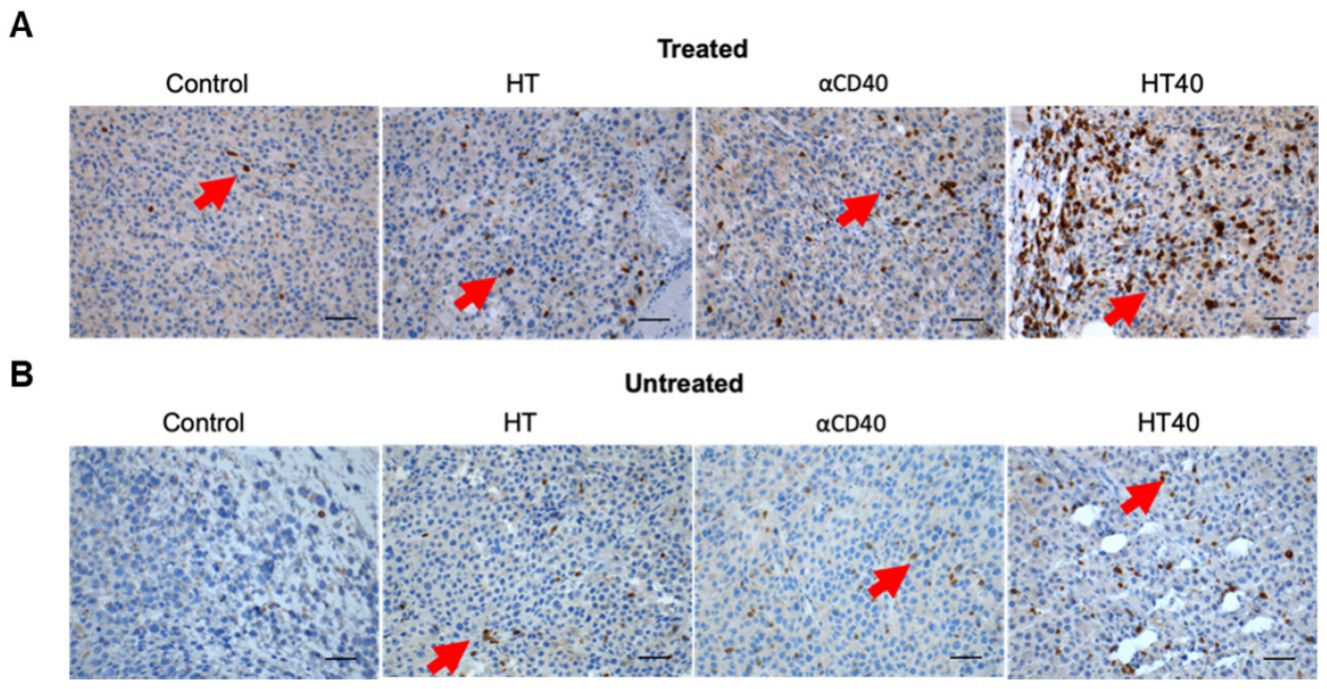
** HT40 therapy enhanced infiltration of T cells in the treated and distant untreated (abscopal) tumors.** (A & B) Representative examples of the immunohistochemical staining of treated (A) and untreated tumor (B) sections for CD3 marker. Both treated and untreated tumors from HT40 cohort showed an increased population of CD3+T cells in the tumor (*arrows*) compared to monotherapies. Scale bar: 100μm.

HT treatment also lowered the immunosuppressive cytokine TGFβ2 in tumors, and the addition of αCD40 caused upregulation of several immune-activation markers, including CXCL9. Chemokines such as CCL3-5, CCL8, CCL11-12, CXCL9 and CXCL10 produced from mature APCs play a crucial role in recruiting CD8+ T cells, CD4+ helper T cells, and natural killer cells into TME 64, 66. CXCL9 also positions tumor infiltrating T cells in APC rich regions to remove T cell anergy 67. CXCL9 is constitutively produced from myeloid cells following stimulation of IFN secreting T cells 67, 68. IFN-γ can induce additional production of this chemokines via STAT1 signaling to enhance CD8+ T cells recruitment into tumors 69-71. Our tumor immune analysis suggested that HT40 treatment induced an influx of CD8+ IFN-γ expressing T cells (Figure 5 and 6), indicating a CXCL9 mediated amplification of cytotoxic T cell-based antitumor immunity 72, 73. In addition, increased accumulation of M1 macrophages and granzyme B+ activated CD8+ T cells without alteration of Tregs was noted in tumors treated with HT40 (Figure 7). Also, the population of TRP-2 specific CD4+ T cells and CD44hi CD62lo CD4+ T cells that help with the memory T cell response was enhanced. These were also verified in IHC analysis where relatively enhanced populations of CD3+ T cells were observed in the treated and untreated tumors of HT cohorts (Figure 10). HT also increased PD-L1, CTLA4, and other immune checkpoints within the tumor microenvironment (Figure 3). These phenotypic alterations are typically an adaptive mechanism to suppress T cell function 74. However, enhanced expression of checkpoint proteins can also be a positive prognostic marker of ICI outcomes in melanoma patients 75-77. To investigate whether this was true in our model system, ICIs were added to the HT40 regimen, and this resulted in improved efficacy and mice survival rates (Figure 8 and 9). Thus, we believe that HT40 may have significant clinical value, especially when combined with ICIs or other immune activators such as TLR and chemokine/cytokine agonists.

Our study had some limitations. First, HT40 therapy improved survival but did not eliminate the melanoma tumors. We do not know the reasons for this outcome, but the response of melanoma to HT40 may depend on the degree of mechanical damage, dosing, sequence, and schedule of the HT and αCD40 therapies. Studies are currently underway to further investigate these mechanisms. These include first enhancing CD40 stimulation in smaller tumors, followed by HT40 treatment of larger tumors to provide sufficient priming. Alternatively, combining FUS parameters (e.g., mild hyperthermia + HT) with CD40 stimulation might be more insightful. Second, although the addition of HT40 to ICI improved the response of refractory melanoma, local recurrence and emergence of distant metastasis may still be possible 78. Future re-challenge studies and histopathological evaluations of lung tissues may shed more light on such mechanisms. Third, only a single B16F10 model and single HT40 treatment was investigated. Future studies employing multiple models with multiple HT40 doses would elucidate the differences in clinical efficacies of various therapies. Lastly, mechanical homogenization of tumors using HT can induce metastasis. This aspect was not studied, although recent studies from other groups suggest that it is highly unlikely 27, 29.

In summary, boiling HT40 therapy augmented innate and adaptive immunity in the B16F10 model. An inflamed TME with an active interaction of CXCL9-cytotoxic T cell axis was the likely mechanism responsible for sensitization to ICI and improved survival rates of mice. Combining HT40 with ICIs may enhance outcomes in advanced stage cancer patients.

## Supplementary Material

Supplementary figures.
