# Supplementary material for: Boiling histotripsy and in-situ CD40 stimulation improve the checkpoint blockade therapy of poorly immunogenic tumors
Source: Theranostics. 2021 Jan 1;11(2):540–54. doi: 10.7150/thno.49517 (PMC7738858; doi:10.7150/thno.49517)
Supplement: Supplementary file 1 — Supplementary figures. [file thnov11p0540s1.pdf]

Supplementary Information

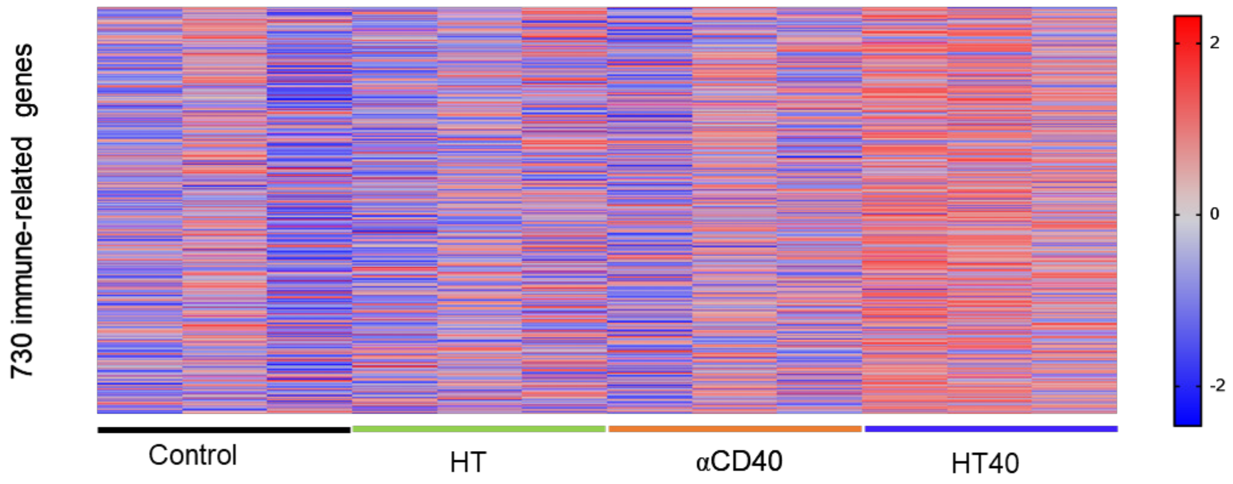

Figure S1. Pan cancer immune profiling by nanostring analysis assessed 730 immuno regulatory genes in the treated tumors (n = 3 per group).

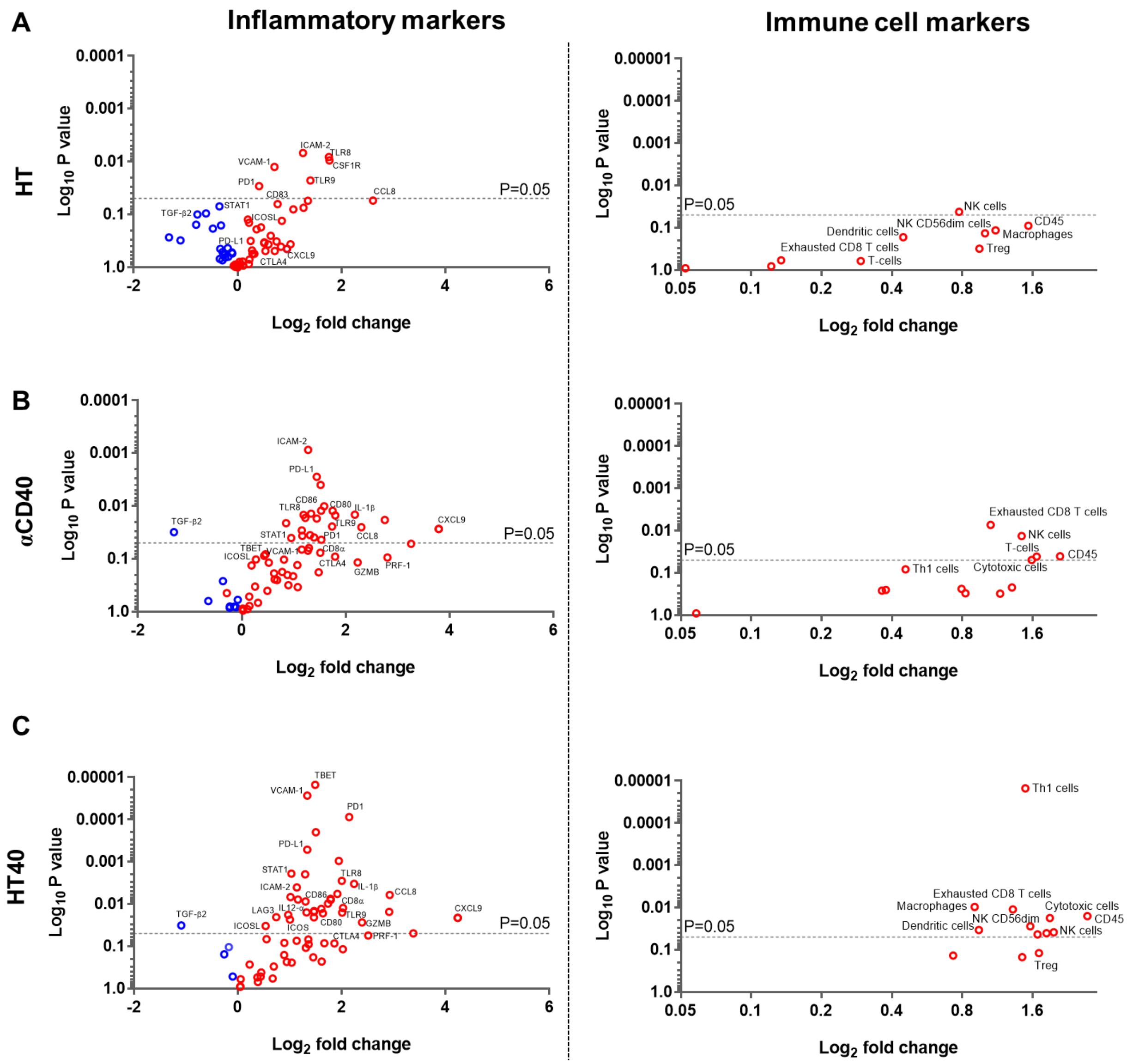

Figure S2. Quantitative assessment of inflammatory and immune cell markers. (A-C) Significantly higher expression of cell adhesion molecules, chemokines, innate sensors, activation status of APCs, natural killer cells (NK), and T cells was noted in HT40 tumors relative to control. The volcano plots represent log2 fold change in gene expression compared to control. Statistical analysis was performed using multiple t-tests without multiple comparisons correction. $p < 0.05$ is considered significant.
